# Acid–base changes after fluid bolus: sodium chloride vs. sodium octanoate

**DOI:** 10.1186/2197-425X-1-4

**Published:** 2013-10-29

**Authors:** Lu Ke, Paolo Calzavacca, Michael Bailey, Wei-qin Li, Rinaldo Bellomo, Clive N May

**Affiliations:** Howard Florey Institute, University of Melbourne, Parkville, Melbourne, Victoria 3052 Australia; Surgical ICU, Department of General Surgery, Jinling Hospital, Nanjing, 210002 China; School of Medicine, Nanjing University, Nanjing, Jiangsu 210093 China; Department of Intensive Care, Austin Health, Heidelberg, Victoria 3084 Australia; Department of Medicine, Austin Health, Heidelberg, Victoria 3084 Australia; Department of Anaesthesia and Intensive Care, AO Melegnano, PO Uboldo, Cernusco sul Naviglio, Italy; Australian and New Zealand Intensive Care Research Centre, Monash University, Melbourne, Victoria, 3800 Australia; Intensive Care Unit, Austin Health, 145 Studley Road, Heidelberg, Victoria, 3084 Australia

**Keywords:** Sodium chloride, Normal saline, Sodium octanoate, Metabolic acidosis, Strong ion difference

## Abstract

**Objectives:**

This study aims to test the hypothesis that fluid loading with sodium chloride (150 mmol Na and 150 mmol Cl) or sodium octanoate (150 mmol Na, 100 mmol Cl, and 50 mmol octanoate) would lead to different acid–base changes.

**Design:**

We performed a double-blind crossover experimental study.

**Setting:**

The study was done at a University Physiology Laboratory.

**Subjects:**

Eight Merino ewes were used as subjects.

**Measurements and main results:**

We randomly assigned animals to a rapid intravenous infusion (1 L over 30 min) of either normal saline (NS) or sodium octanoate solution (OS). We collected blood samples at 0.5, 1, 2, 4, and 6 h after the start of the infusion for blood gas analyses and biochemistry. We calculated strong ion difference apparent (SIDa), effective strong ion difference, and strong ion gap (SIG). Animals in the NS group developed metabolic acidification immediately after fluid administration (pH 7.49 to 7.42, base excess 3.0 to -1.6 mEq/L), while the OS group did not (pH 7.47 to 7.51, base excess 1.1 to 1.4 mEq/L; *P* < 0.001). Additionally, the OS group had higher SIDa (36.2 vs. 33.2 mEq/L) and SIG (7.4 vs. 6.2 mEq/L) at the end of the infusion.

**Conclusions:**

Our findings provide further evidence that acidification induced by intravenous fluid loading is dependent on fluid composition and challenges the paradigm of the so-called *dilutional* acidosis.

**Electronic supplementary material:**

The online version of this article (doi:10.1186/2197-425X-1-4) contains supplementary material, which is available to authorized users.

## Background

Large-volume infusion of normal saline (NS) has been repeatedly associated with metabolic acidosis [1–4]. The causal mechanism for this post-infusion acidosis has been repeatedly ascribed to the dilution of serum buffers and has, therefore, been named 'dilutional acidosis’ [5–7]. However, the correctness of this view has been challenged by the lack of significant acid–base change after infusion of equivalent volumes of so-called balanced fluids such as Ringer's lactate, sodium acetate, and other solutions [3, 8, 9] and the emergence of the Stewart approach to acid–base physiology. This approach argues that a change in the strong ion difference (SID) [10, 11] is most likely responsible for the acidosis of saline administration.

This controversy is difficult to resolve because balanced fluids with an *in vivo* physiological SID contain substances like lactate or acetate or gluconate, which are believed to immediately enter the citric acid cycle and become a source of bicarbonate. Such bicarbonate can then allegedly compensate for the 'dilutional effect’ of volume expansion and prevent acidosis. However, a strong ion, which is biologically safe, does not immediately generate bicarbonate and might help elucidate whether the 'dilutional’ acidosis paradigm is correct. Octanoate might be such a strong ion.

Octanoate is a medium-chain fatty acid (MCFA) with a pK of 4.89 found in milk and tropical plants. It can be made soluble and used as an anion to replace some of the chloride found in saline. Additionally, octanoate's metabolism is by beta-oxidation making it unlikely that it would have any immediate impact on bicarbonate generation. If the acidosis of saline were truly due to dilution, there should be no differences in the acid–base effects of the two solutions. If, instead, the acidosis were due to differences in the SID, then the changes in acid–base balance should differ between the two fluids. We performed a randomized, double-blind, crossover animal experiment to test the hypothesis that sodium octanoate would not lead to the same acidification seen with normal saline.

## Methods

### Animal preparation

This study was approved by the Animal Experimentation Ethics Committee of the Howard Florey Institute, Melbourne, Australia. Experiments were performed on eight conscious adult Merino ewes weighing between 25 to 40 kg, housed in individual metabolic cages.

On the day prior to the experiment, an arterial Tygon catheter and internal jugular venous polythene catheter were inserted for blood sampling and fluid infusion, respectively.

### Experimental protocol

Animals were randomly assigned to either sodium chloride (150 mmol Na and 150 mmol Cl) or sodium octanoate (150 mmol Na, 100 mmol chloride, and 50 mmol octanoate). The solutions were provided in indistinguishable glass bottles prepared for the experiment by CSL Bioplasma (CSL, Melbourne, Australia). Thus, assignment was random and concealed, and the investigator infusing the fluid was blinded to the type of fluid. The following day, the animal was assigned to the other fluid in a crossover design, such that those animals first assigned to sodium chloride then received sodium octanoate the next day and those first assigned to sodium octanoate then received sodium chloride the next day.

Baseline blood samples were taken for blood gas analysis (ABL800 Blood Gas Analyzer, Radiometer, Copenhagen, Denmark) and measurement of serum magnesium, phosphate, and albumin levels (SYNCHRON LX® System Beckmann Coulter Inc., Fullerton, CA, USA). After such baseline measurements, animals received a rapid intravenous infusion (over 30 min) of 1 L of trial solution. Additional blood samples were then collected at 0.5, 1, 2, 4, and 6 h after the start of the infusion.

### Calculations for the interpretation of quantitative acid–base analysis

Quantitative biophysical analysis of the results was performed with the Stewart approach [10] as modified by Figge et al. [11] to take into account the effects of other factors like albumin level. This method first calculates the apparent strong ion difference (SIDa):

$$\begin{array}{c} \mathrm{SIDa}=\left[{\mathrm{Na}}^{+}\right]+\left[K^{+}\right]+\left[{\mathrm{Mg}}^{2+}\right]+\left[{\mathrm{Ca}}^{2+}\right]‒\left[{\mathrm{Cl}}^{‒}\right]‒\left[\mathrm{Lactate}\right]\\ \left(\mathrm{all}\;\mathrm{concentrations}\;\mathrm{in}\;\mathrm{mEq}/L\right) \end{array}$$

However, the role of weak acids (carbon dioxide, albumin, and phosphate) in the balance of electrical charges in plasma water is not taken into account in this equation. The effective strong ion difference (SIDe) was thus calculated according to Figge et al. [11] as follows:

$$\begin{array}{c} \mathrm{SIDe}=2{.46\times 10}^{\left(\mathrm{pH}-8\right)}\times p{\mathrm{CO}}_{2}+\left[\mathrm{Albumin}\right]\times \left(0.12\times \mathrm{pH}-0.631\right)+\left[\mathrm{Phosphate}\right]\times \left(0.309\right)\times \mathrm{pH}-0.469.\\ \left(p{\mathrm{CO}}_{2}\;\mathrm{in}\;\mathrm{mmHg},\mathrm{albumin}\;\mathrm{in}\;g/L,\mathrm{and}\;\mathrm{phosphate}\;\mathrm{in}\;\mathrm{mmol}/l\right) \end{array}$$

Without unmeasured charges, SIDa - SIDe should be equal to zero (electrical charge neutrality) once weak acids are quantitatively taken into account. If a gap exists between SIDa and SIDe, then unmeasured anions (e.g., sulfate, keto acids, citrate, pyruvate, acetate, and gluconate) must be present to explain this gap which is termed the strong ion gap (SIG): SIG = SIDa - SIDe.

### Statistical analysis

To ascertain if NS was significantly different from octanoate solution (OS) over the 6-h period or if NS behaved differently over time, we used repeated measures analysis of variance model fitting main effects for group (NS or OS), time (two times using 0 and 6 h), and an interaction between group and time to determine if the two groups behave differently over time. Modeling was performed using the PROC Mixed procedure in SAS version 9.2 (SAS Institute Inc., Cary, NC, USA) with the six individual sheep treated as random effects. Nine outcome variables were considered (pH, base excess, hematocrit, sodium, chloride, bicarbonate, SIDa, SIDe, SIG), meaning nine models were constructed. Results have been reported as least square means (95% confidence intervals).

## Results

### Changes in pH, base excess, and hematocrit

**Figure 1:**
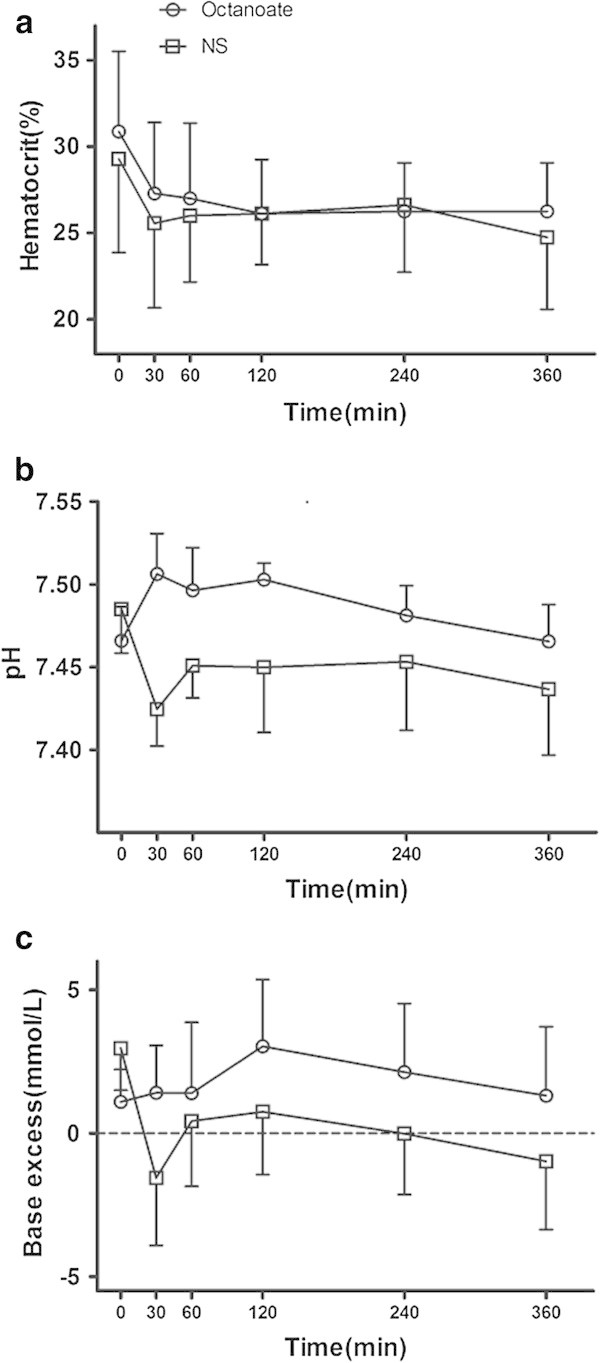
**Hematocrit (a), pH (b), and BE (c) for NS and OS groups during the study period.**

After infusion of sodium chloride, both pH and base excess (BE) decreased significantly and remained below the baseline value for the duration of the study. In contrast, there was a slight increase in pH and BE after sodium octanoate infusion (Figure 1b,c, Tables 1 and 2). Hematocrit decreased in both groups, indicating hemodilution. However, no statistical difference in this indicator of dilution could be detected between the two groups throughout the experiment (Figure 1a, Tables 1 and 2).

**Table 1:** **Differences in hematocrit, pH, and BE when comparing octanoate with normal saline after fluid infusion**

Variable	Group	DFB	DFBstderr	Probt
Hematocrit (%)	Octanoate	-4.343	0.730	0.055
	Normal saline	-3.400	0.746	
pH	Octanoate	0.024	0.006	*P* < 0.001
	Normal saline	-0.037	0.006	
BE (mmol/L)	Octanoate	0.723	0.374	*P* < 0.001
	Normal saline	-2.951	0.385	

BE, base excess; DFB, difference from baseline; DFBstderr, standard error of the difference from baseline; Probt, significance of overall difference.

**Table 2:** **Variable differences using RM-ANOVA model fitting main effects for group, time, and group-time interaction**

Variable	Question	Test	***P*** value
Hematocrit	Is there an overall difference between saline and octanoate over the 6-h observation period in healthy conscious sheep?	Group effect RM-ANOVA	0.055
	Does octanoate behave differently from saline over time in the 6-h observation period in healthy conscious sheep?	Interaction between group and time RM-ANOVA	0.479
pH	Is there an overall difference between saline and octanoate over the 6-h observation period in healthy conscious sheep?	Group effect RM-ANOVA	<0.001
	Does octanoate behave differently from saline over time in the 6-h observation period in healthy conscious sheep?	Interaction between group and time RM-ANOVA	0.011
BE	Is there an overall difference between saline and octanoate over the 6-h observation period in healthy conscious sheep?	Group effect RM-ANOVA	<0.001
	Does octanoate behave differently from saline over time in the 6-h observation period in healthy conscious sheep?	Interaction between group and time RM-ANOVA	0.370

BE, base excess; RM-ANOVA, repeated measures analysis of variance. Variables (hematocrit, ph, BE), group (saline and octanoate), and time (0 to 6 h). Because of multiple comparisons, only *P* < 0.01 was considered statistically significant.

### Changes in electrolytes

**Figure 2:**
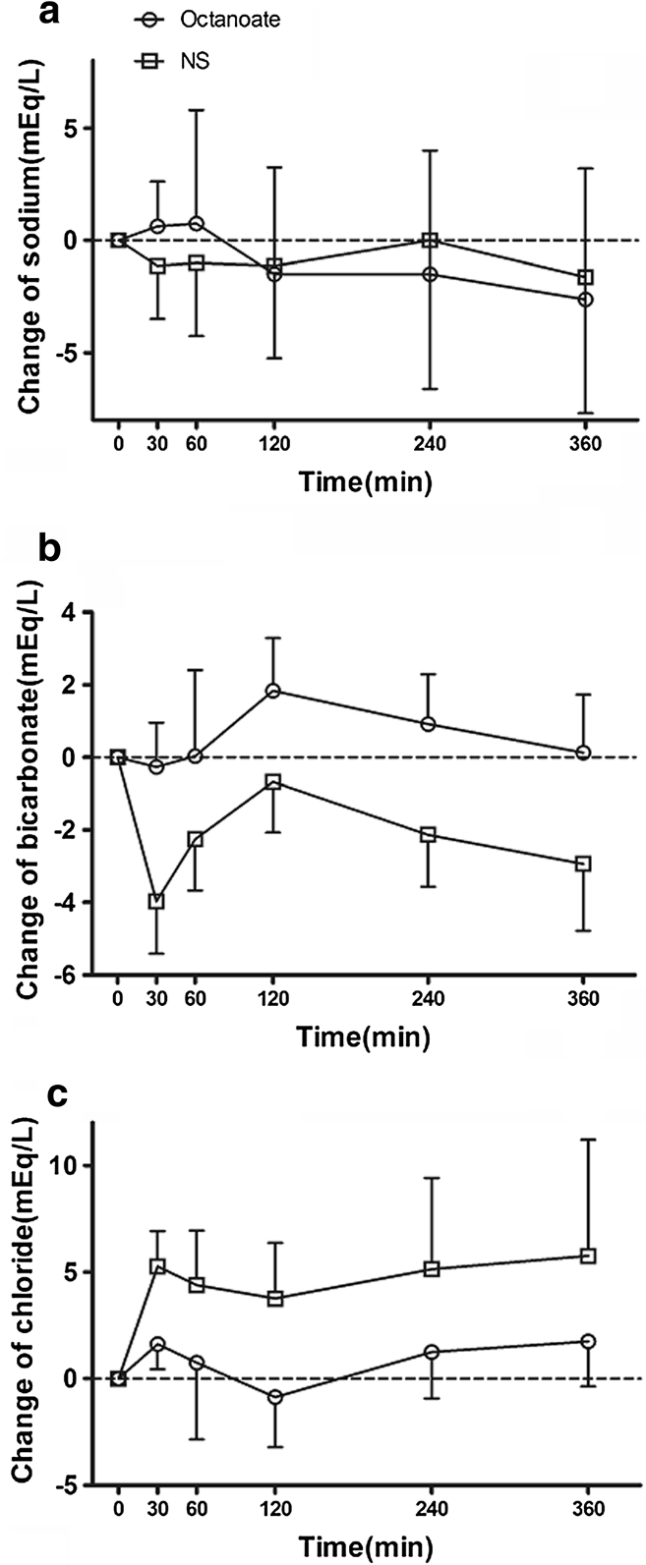
**Electrolytes in the normal saline and octanoate groups during the study period. (a)** Change in sodium, **(b)** change in bicarbonate, and **(c)** change in chloride.

Figure 2 shows the change in sodium, bicarbonate, and chloride levels from baseline values. Sodium levels in both experimental groups remained unchanged after infusion. In contrast, bicarbonate and chloride levels evolved differently between the two groups (Figure 2a, Table 3). The sodium chloride group showed a significant increase in chloride and decrease in bicarbonate levels after fluid challenge, while there was a slight increase in bicarbonate and no change in chloride after octanoate infusion (Figure 2b,c, Table 3).

**Table 3:** **Differences in electrolyte changes using RM-ANOVA model fitting main effects for group, time, and group-time interaction**

Variable	Question	Test	***P*** value
Change in sodium	Is there an overall difference between saline and octanoate over the 6-hobservation period in healthy conscious sheep?	Group effect RM-ANOVA	0.830
	Does octanoate behave differently from saline over time in the 6-hobservation period in healthy conscious sheep?	Interaction between group and time RM-ANOVA	0.800
Change in bicarbonate	Is there an overall difference between saline and octanoate over the 6-h observation period in healthy conscious sheep?	Group effect RM-ANOVA	<0.001
	Does octanoate behave differently from saline over time in the 6-h observation period in healthy conscious sheep?	Interaction between group and time RM-ANOVA	0.004
Change in chloride	Is there an overall difference between saline and octanoate over the 6-h observation period in healthy conscious sheep?	Group effect RM-ANOVA	<0.001
	Does octanoate behave differently from saline over time in the 6-h observation period in healthy conscious sheep?	Interaction between group and time RM-ANOVA	0.147

Variables (change in sodium, bicarbonate, chloride), group (saline and octanoate), and time (0 to 6 h).Because of multiple comparisons, only *P* < 0.01 was considered statistically significant.

### Stewart analysis

**Figure 3:**
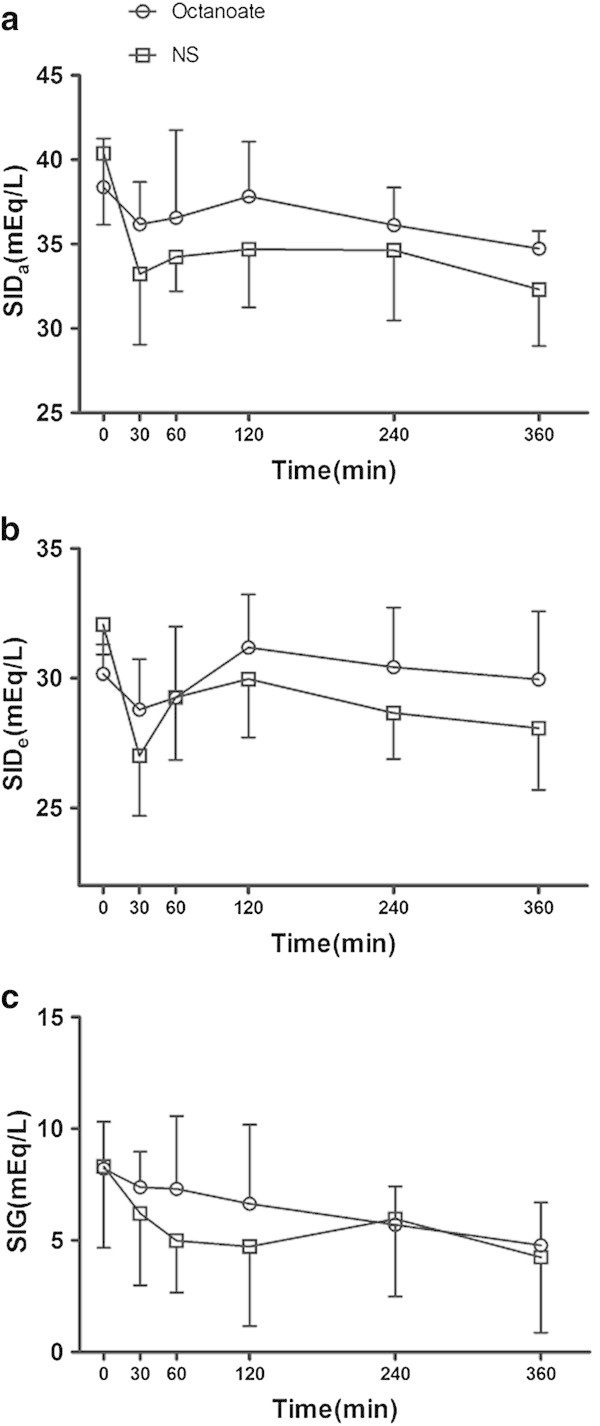
**Stewart's approach parameters in the normal saline and octanoate groups during the study period. (a)** SIDa, **(b)** SIDe, and **(c)** SIG.

At the end of the infusion, the SIDa level in the octanoate group was 36.2 ± 2.5 mEq/L compared with 33.2 ± 4.2 mEq/L in the sodium chloride group. This significant increase in SIDa with octanoate persisted throughout the observation period (Figure 3a, Tables 4 and 5). Regarding SIDe, both groups showed decreased levels initially, but the changes in the two groups were statistically different with better overall preservation with octanoate (Figure 3b, Tables 4 and 5). In addition, the octanoate group showed a slightly higher level of SIG after the infusion suggesting a minor increase in unmeasured anions (Figure 3c, Tables 4 and 5).

**Table 4:** **Differences in Stewart parameters when comparing octanoate with normal saline after fluid infusion**

Variable	Group	DFB	DFBstderr	Probt
SIDa(mEq/L)	Octanoate	-2.157	0.927	*P* < 0.001
	Normal saline	-6.413	0.948	*P* < 0.001
SIDe(mEq/L)	Octanoate	-0.288	0.409	*P* < 0.001
	Normal saline	-3.242	0.422	*P* < 0.001
SIG(mEq/L)	Octanoate	-1.866	0.996	*P* = 0.068
	Normal saline	-3.167	1.019	*P* = 0.068

DFB, difference from baseline; DFBstderr, standard error of difference from baseline; Probt, significance of overall difference; SID, strong ion difference; SIG, strong ion gap.

**Table 5:** **Differences in variables using RM-ANOVA model fitting main effects for group, time, and group-time interaction**

Variable	Question	Test	***P*** value
SIDa	Is there an overall difference between saline and octanoate over the 6-h observation period in healthy conscious sheep?	Group effect RM-ANOVA	<0.001
	Does octanoate behave differently from saline over time in the 6-h observation period in healthy conscious sheep?	Interaction between group and time RM-ANOVA	0.840
SIDe	Is there an overall difference between saline and octanoate over the 6-h observation period in healthy conscious sheep?	Group effect RM-ANOVA	<0.001
	Does octanoate behave differently from saline over time in the 6-h observation period in healthy conscious sheep?	Interaction between group and time RM-ANOVA	0.470
SIG	Is there an overall difference between saline and octanoate over the 6-h observation period in healthy conscious sheep?	Group effect RM-ANOVA	0.068
	Does octanoate behave differently from saline over time in the 6-h observation period in healthy conscious sheep?	Interaction between group and time RM-ANOVA	0.749

SID, strong ion difference; SIG, strong ion gap. Variables (SIDa, SIDe, SIG), group (saline and octanoate), and time (0 to 6 h). Because of multiple comparisons, only *P* < 0.001 was considered statistically significant.

## Discussion

### Key findings

We performed a double-blind, randomized crossover experimental study to compare the acid–base effects of rapid sodium chloride and sodium octanoate infusion. We hypothesized that these two fluids would lead to significantly different acid–base responses despite similar dilutional effects and the absence of a 'buffer’ in the octanoate fluid. In keeping with our hypothesis, we found no significant difference in hematocrit between the two groups after rapid fluid infusion indicating equivalent levels of 'dilution’. Yet, we simultaneously found that NS infusion induced metabolic acidification with a significant decrease in pH, base excess, and bicarbonate while OS did not.

### Relationship to previous studies

Similar differences in the acid–base effects of fluids have been reported when comparing NS and a number of balanced solutions such as Ringer's lactate and Plasma-Lyte [9]. As dilution could not sufficiently explain this phenomenon, some previous studies attributed this difference to hyperchloremia and decreased SID [8, 12, 13], in accordance with Stewart's physicochemical approach. Morgan and colleagues showed that a balanced solution should have a SID of 24 mEq/L to be pH neutral [14]. According to the Stewart approach, chloride is the key contributor to the SID; thus, different amounts of chloride contained in 0.9% saline and balanced solutions are likely of great importance in the pathogenesis of metabolic acidosis. Additionally, it is noteworthy that these balanced solutions have an *in vitro* SID equal to zero, similar to isotonic saline. *In vivo*, however, the metabolism of these anions increases the SID, a key mechanism by which, according to the Stewart paradigm, balanced fluids avoid metabolic acidosis [9].

However, commercially available balanced crystalloid solutions all contain substances that can rapidly generate bicarbonate through the citric acid cycle immediately after infusion [15]. Similarly, acetate could also produce bicarbonate soon after administration through rapid metabolism [16, 17]. Due to these metabolic characteristics, it is impossible to know whether the lack of acidosis seen with the administration of these balanced solutions is due to bicarbonate production or the near-normal or normal SID of the solution.

Octanoate [CH_3_(CH_2_)6CO_2_H] is a medium-chain fatty acid subject to liver clearance. It is widely used for the measurement of gastric emptying rate in breath testing or as a surrogate marker of liver function [18–20]. In hepatocytes, it undergoes beta-oxidation [18, 19, 21]. Thus, the preserved levels of pH, BE, and bicarbonate after fluid challenge in the OS group appear more likely explained by its more physiological *in vivo* SID rather than the rapid generation of bicarbonate; this is in contrast with the decreased pH, BE, and bicarbonate shown in the NS group that could only be logically attributed to reduced SID and hyperchloremia instead of dilution.

A previous study showed that even using a balanced solution, mild acidosis could still be present for a short time after rapid delivery of large amounts of fluid [1, 15]. This effect was associated with an increased SIG which likely reflected a surge in unmeasured anions like gluconate and acetate prior to their metabolism and uptake [1]. Differently, the octanoate group showed similar SIG levels to the saline group and no signs of acidosis in our study, as both pH and BE increased even at the end of the infusion. These results suggest that octanoate can be rapidly removed from the circulation with only very limited effect on SIG.

### Implications for clinicians

Our study provides additional evidence that the mechanism responsible for metabolic acidosis after rapid fluid resuscitation is not dilution but rather the administration of a fluid with a non-physiological SID. This observation should assist in the choice of appropriate fluids for intravenous therapy in different critically ill patients according to their physiological targets and baseline acid–base characteristics. In addition, the introduction of octanoate as a potential ion to be used in the development of targeted optimal *in vivo* SID intravenous fluids is of interest. Octanoate is already used in specific albumin preparations used in Australia and New Zealand at concentrations varying from 6 to 32 mmol/L and has therefore already been given to thousands of patients without evidence of adverse effects.

### Strengths and limitations

This is a double-blind randomized controlled experimental study. It shows clear differences in the physiological outcomes of interest and provides novel evidence to challenge the notion that dilution is responsible for fluid-associated acidosis. The data are objective, measured by standard technology, and the robustness of the findings is strong. However, our findings do not provide evidence to demonstrate any clinical benefit in association with the prevention of saline-associated acidosis. Human studies are required to demonstrate such potential benefits if they exist. It is possible that octanoate was rapidly transformed into bicarbonate and thus acted in a manner similar to lactate- or acetate-buffered solutions. Although this may have occurred at a later time, the lack of any changes in serum bicarbonate at 30 and 60 min and knowledge about the pathway for octanoate metabolism strongly suggests that the changes in acid–base status seen in the first hour cannot logically be explained by octanoate-related bicarbonate generation. Other explanations can be offered for our findings. Octanoate, a MCFA, can penetrate the cell without impairment and be metabolized rapidly in the cytoplasm. As a MCFA, it can penetrate the mitochondrial membrane without the need for carnitine palmitoyl transferase I. Within the mitochondrial matrix, it is metabolized via beta-oxidation into either AcCoA with consumption of oxygen and generation of CO_2_ via the citric acid cycle, with generation of ATP and consumption of H^+^, or - in the absence of insulin and the presence of high glucagon - into ketone bodies, with generation of metabolic acidosis [22]. However, β-oxidation does not necessarily lead to ketoacidosis. Assuming stable nutrition and adequate oxygenation, most of the octanoate infused may have been rapidly β-oxidized to fatty acyl-CoA and eventually acetyl-CoA leading to the generation of ATP via phosphorylative oxidation with consumption of 2H^+^, thus preventing acidosis or leading to alkalosis.

## Conclusions

In conclusion, our findings provide further evidence that acidification induced by saline infusion can be explained by changes in SID. Once a solution is given with a chloride substitute (octanoate), which can be removed rapidly from the circulation, acidification is prevented. The acidosis of saline resuscitation cannot be logically ascribed to dilution.

## Authors’ original submitted files for images

Below are the links to the authors’ original submitted files for images. Authors’ original file for figure 1 Authors’ original file for figure 2 Authors’ original file for figure 3
