# Supplementary figures and images for: Acid–base changes after fluid bolus: sodium chloride vs. sodium octanoate
Source: Intensive Care Med Exp. 2013 Oct 29;1:4. doi: 10.1186/2197-425X-1-4 (PMC4797930; doi:10.1186/2197-425X-1-4)

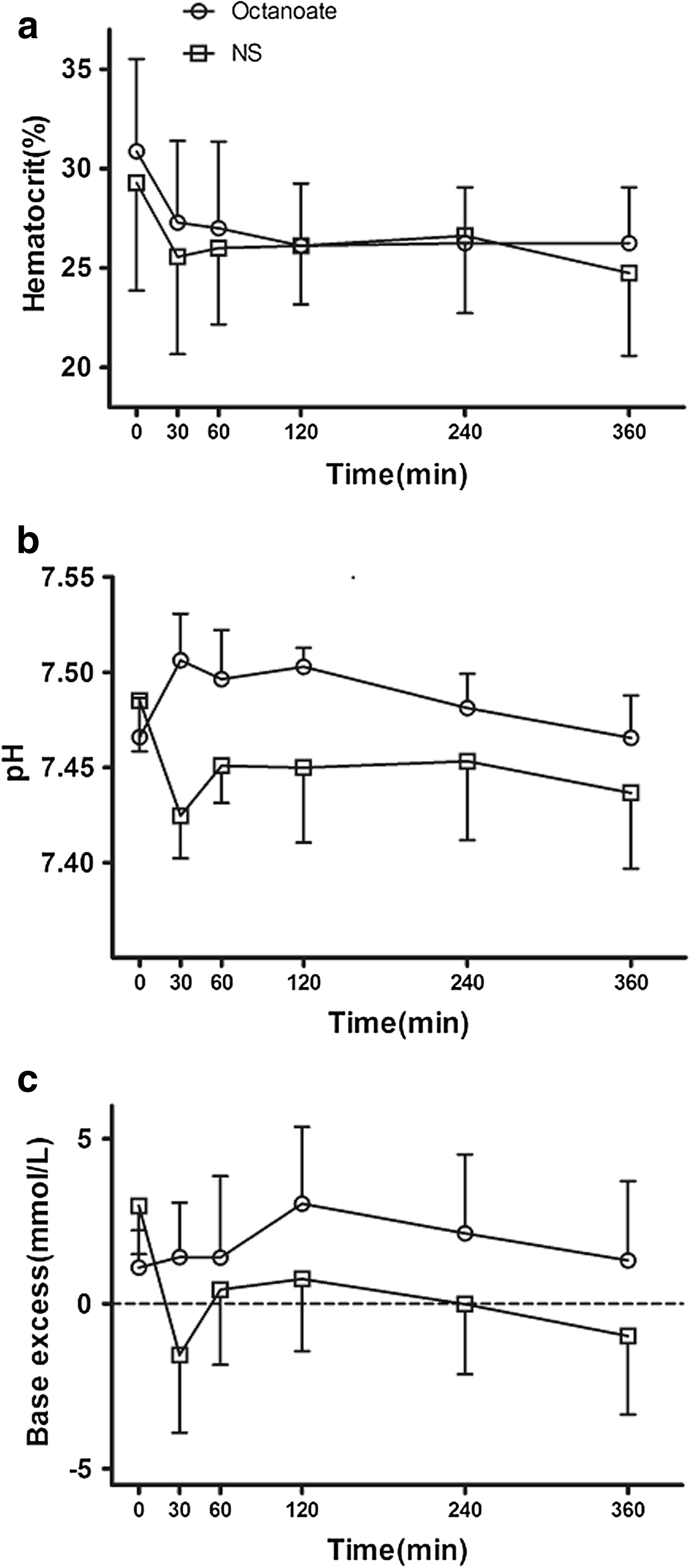

Supplement: Supplementary file 1 — Authors’ original file for figure 1 [file 40635_2013_23_MOESM1_ESM.tiff]

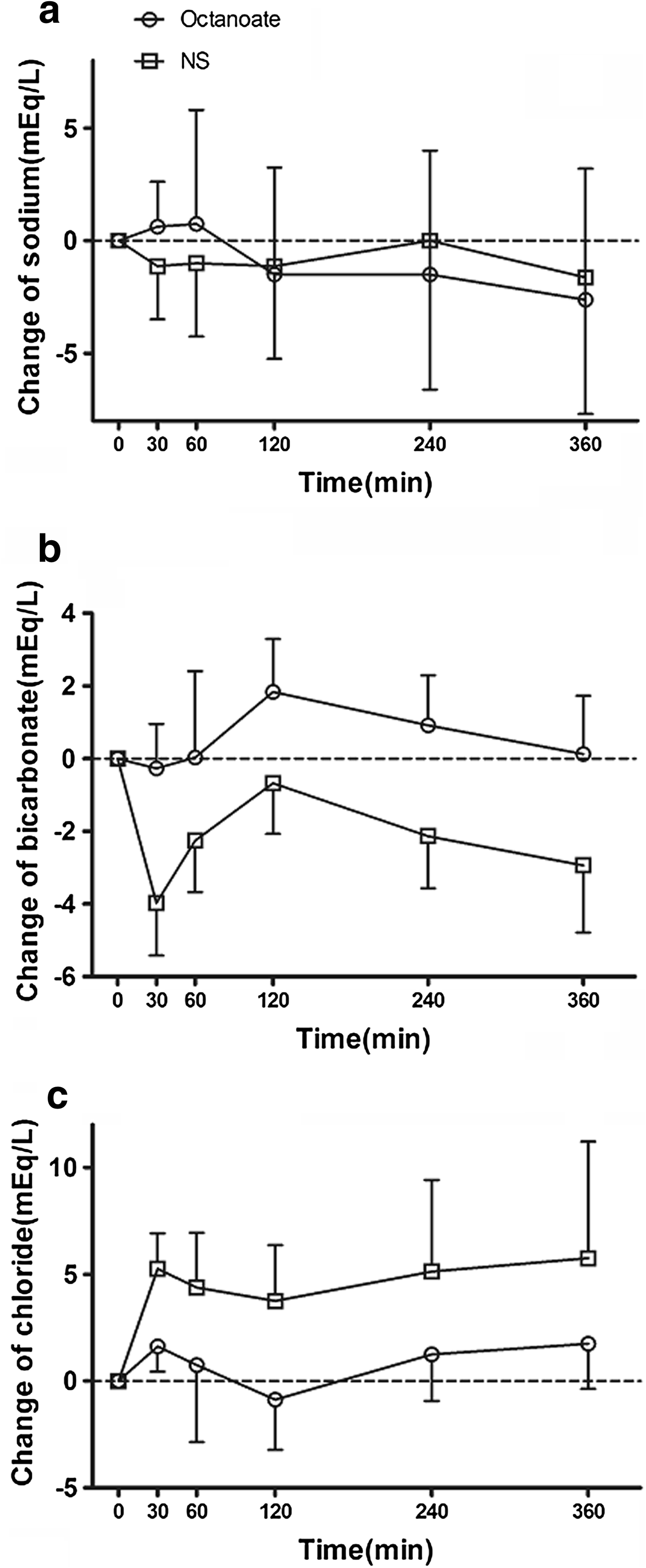

Supplement: Supplementary file 2 — Authors’ original file for figure 2 [file 40635_2013_23_MOESM2_ESM.tiff]

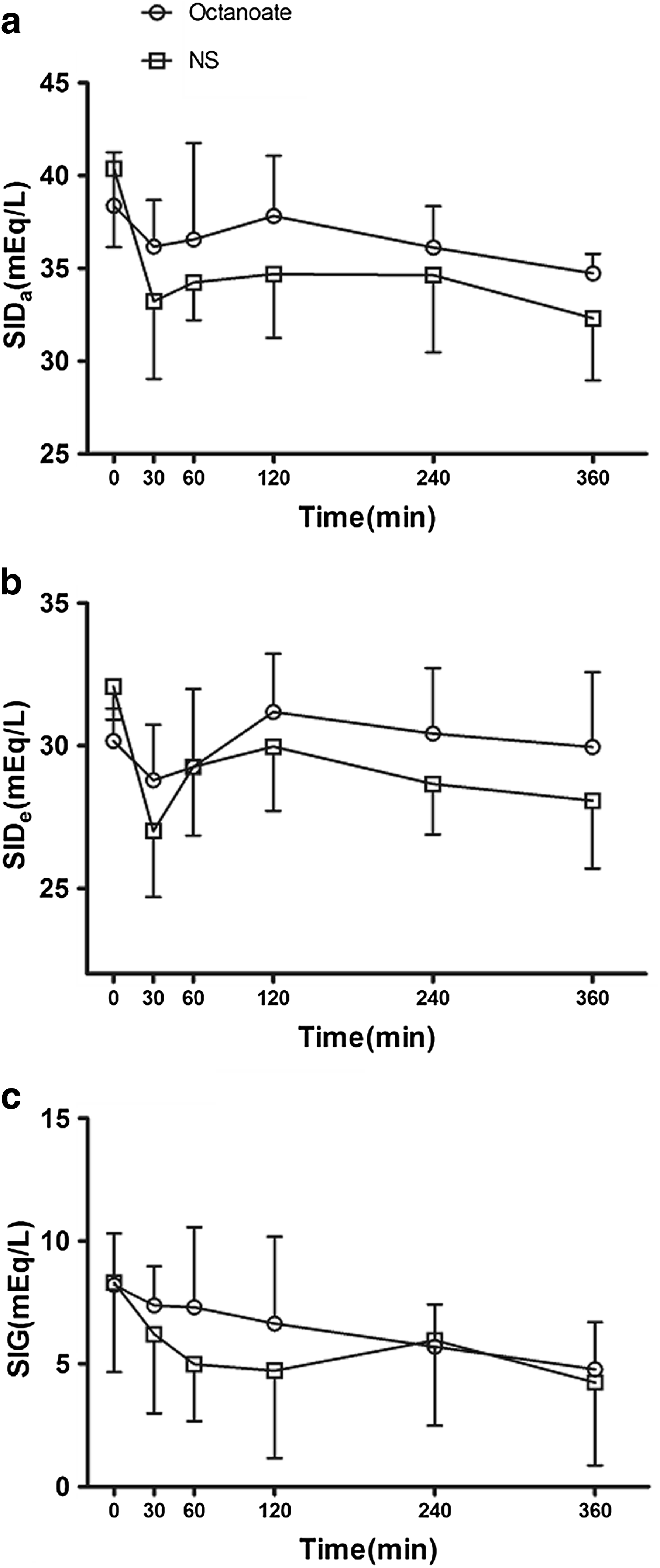

Supplement: Supplementary file 3 — Authors’ original file for figure 3 [file 40635_2013_23_MOESM3_ESM.tiff]
